# A Practical Treatise on Diseases of the Skin

**Published:** 1898-02

**Authors:** 


A Practical Treatise on Diseases of the Skin. By John V. Shoemaker, M. D., LL. D., Professor of Skin and Venereal Diseases in the Medico-Chirurgical College and Hospital of Philadelphia ; Physician to the Philadelphia Hospital for Diseases of the Skin ; Member of the American Medical Association ; of the Pennsylvania and Minnesota State Medical Societies ; of the American Academy of Medicine, and of the British Medical Association ; Fellow of the Medical Society of London. Third edition, revised and enlarged with chromogravure plates and other illustrations. New York : D. Appleton & Company. 1897.

This large and elaborate work, which constitutes the third edition of the author's Practical treatise of diseases of the skin, contains an enormous amount of well-arranged information regarding skin affections. When we point out that the volume consists of a well-bound book of some 894 pages, a fair notion may be obtained of the bulk of the work and the labor expended in its production.

The treatise has been planned with certain limitations, and is especially devoted to the consideration of the symptomatology, diagnosis, etiology and treatment. The author has happily avoided historical sketches, solely treating the subject matter from the standpoint of the general pathologist and, furthermore, he has emphasised the most notable recent views of the various writers upon these topics which have commended themselves as of practical value.

The classification adopted is a modification of the one promulgated by Hebra, including disorders of secretion and excretion, hyperemias, hemorrhages, hypertrophies, atrophies, tumors, neuroses and parasites. The anatomy and physiology of the skin and its appendages is considered in thirty-seven pages, thoroughly worked up to date and freely illustrated. Another feature, which considerably increases the number of pages, is a valuable and extensive formulary in the concluding portion of the volume.

The illustrations, although not profuse, on the whole are good and the colored plates of psoriasis, dermatitis venenata, and alopecia circumscripta are decidedly admirable. The work will prove of value both to the advanced student and to the practitioner who wishes to renew his acquaintance with dermatology from the modern standpoint. E. W.

